# Antitumor effects of the GM3(Neu5Gc) ganglioside-specific humanized antibody 14F7hT against *Cmah*-transfected cancer cells

**DOI:** 10.1038/s41598-019-46148-1

**Published:** 2019-07-09

**Authors:** Denise Dorvignit, Kayluz F. Boligan, Ernesto Relova-Hernández, Marilyn Clavell, Armando López, Mayrel Labrada, Hans-Uwe Simon, Alejandro López-Requena, Circe Mesa, Stephan von Gunten

**Affiliations:** 1 Immunobiology Division, Center of Molecular Immunology, Havana, 11600 Cuba; 2 Institute of Pharmacology, University of Bern, Bern, 3010 Switzerland; 3 Animal House Department, Center of Molecular Immunology, Havana, 11600 Cuba; 4 Present Address: Ablynx, Technologiepark 21, 9052 Zwijnaarde, Belgium

**Keywords:** Immunization, Cancer models

## Abstract

The GM3(Neu5Gc) ganglioside represents a tumor-specific antigen that is considered a promising target for cancer immunotherapy. We previously demonstrated that the humanized antibody 14F7hT, specific for this ganglioside, exhibited significant antitumor effects in preclinical hematological tumor models. As this antibody recognizes human tumor tissues from several origins, we addressed its potential effect on different tumor types. The use of cell lines for testing GM3(Neu5Gc)-targeting strategies, in particular for human malignancies, is complicated by the absence in humans of functional cytidine monophospho-*N*-acetyl-neuraminic acid hydroxylase (CMAH), the enzyme required for Neu5Gc sialic acid biosynthesis. Quantitative flow cytometry revealed the absence of surface GM3(Neu5Gc) in several human but also mouse cell lines, in the last case due to low expression of the enzyme. Hypoxia-induced expression of this ganglioside on human SKOV3 cells was observed upon culture in Neu5Gc-containing medium without evidence for CMAH-independent biosynthesis. However, only transfection of the mouse *Cmah* gene into human SKOV3 and mouse 3LL cells induced a stable expression of GM3(Neu5Gc) on the cancer cell surface, resulting in effective models to evaluate the antitumor responses by 14F7hT *in vitro* and *in vivo*. This antibody exerted antibody-dependent cell-mediated cytotoxicity (ADCC) and *in vivo* antitumor effects on these *Cmah*-transfected non-hematological tumors from both mouse and human origin. These results contribute to validate GM3(Neu5Gc) as a relevant target for cancer immunotherapy and reinforces the value of 14F7hT as a novel anti-cancer drug.

## Introduction

For successful cancer immunotherapy, the nature of the targeted tumor-associated antigen is of critical importance. Ideally, the tumor antigen is highly and selectively expressed in cancer cells, thus permitting effective immune responses in absence of immune-related adverse events due to off-tumor effects. Gangliosides, which are glycosphingolipids containing sialic acid residues, are considered interesting therapeutic targets due to their differential expression in malignant tissues^1–3^. Due to the genetic inactivation of the cytidine monophospho-*N*-acetyl-neuraminic acid hydroxylase (CMAH)^4,5^, which is responsible for the conversion of the sialic acid *N*-acetyl-neuraminic acid (Neu5Ac) to *N*-glycolyl-neuraminic acid (Neu5Gc), human healthy tissues predominantly express Neu5Ac-sialoconjugates. In contrast, Neu5Gc-sialoconjugates are often highly expressed in human tumors^6–8^, a phenomenon that has been attributed to the altered metabolic properties of cancer cells with enhanced uptake and incorporation of nonhuman Neu5Gc from Neu5Gc-containing foods, such as red meat^9–11^. To which extent proposed CMAH-independent, alternate biosynthetic pathways also contribute to Neu5Gc expression in cancer cells in patients^12–14^, remains to be explored.

Of particular interest for immunotherapy is the GM3(Neu5Gc) ganglioside, which was undetectable in human healthy tissues^15^, yet is highly expressed in a broad range of human malignancies, including lung, breast and gastrointestinal cancers, or melanoma^15–19^. Notably, the expression of GM3(Neu5Gc) has been associated with poor prognosis in colon and lung cancer^18,19^. Given its tumor-associated expression, GM3(Neu5Gc)-targeting antibodies have been developed^3,20^, including the mouse 14F7 monoclonal antibody^21^, and its humanized variant 14F7hT^22^, for which promising antitumor effects were demonstrated using *in vitro* and *in vivo* models^22,23^. However, these experiments were restricted to the use of only two mouse cell lines, P3X63 myeloma and L1210 lymphocytic leukemia cells, but not other mouse cell lines, which eventually express lower CMAH enzyme levels^24^, or human cell lines with inactive CMAH.

Further uncertainties arise on the ground of the complex sialic acid metabolism in cancer cells. For instance, while hypoxia was shown to increase the uptake and metabolic incorporation of Neu5Gc from culture medium by the upregulation of the sialic acid transporter, sialin^25^, in a recent article it was hypothesized that the enhanced GM3(Neu5Gc) expression under hypoxic conditions might be linked to CMAH-independent, alternate biosynthetic pathways in human cancer cells^12^. However, few human cells lines, including the WERI-Rb-1 and Y79 retinoblastoma cell lines^26^, the non-commercialized ME melanoma cell line^27^ and, very recently, the T24 human bladder cancer cell line^28^, have been reported to express GM3(Neu5Gc), as assessed by staining with 14F7.

On this background, here we screened mouse and human cell lines of different tissue origin for surface expression of GM3(Neu5Gc) using quantitative analysis by flow cytometry. Using an engineered version of 14F7hT, called 7C1 antibody^29^, which recognizes both GM3(Neu5Gc) and GM3(Neu5Ac), and is thus useful for differential staining in combination with 14F7hT, we demonstrated expression of the latter ganglioside in these cell lines. With the exception of P3X63 and L1210, which are known to express GM3(Neu5Gc) on the cell surface^22,30^, the other investigated mouse cell lines were negative for the expression of cell surface GM3(Neu5Gc), which corresponded with low intracellular CMAH protein levels. In line with the human-specific genetic inactivation of CMAH and in contradiction with previous reports^26,31–33^, no GM3(Neu5Gc) surface expression was detected in human cell lines.

In order to evaluate the antitumor effects of 14F7hT, we decided to generate GM3(Neu5Gc)-expressing model cell lines, either by culturing the cells under hypoxic conditions^12,25^ or by transfecting the mouse *Cmah* gene^25,34^. Hypoxia has been described to promote Neu5Gc-ganglioside expression^12,25^. Hypoxia-induced GM3(Neu5Gc) surface expression was detected upon culture of human SKOV3 cells in Neu5Gc-rich fetal bovine serum (FBS), but not Neu5Gc-low human serum (HS), indicating a role of enhanced uptake and metabolic incorporation, in absence of CMAH-independent, alternate biosynthetic pathways. However, stable GM3(Neu5Gc) surface expression was only achieved in human SKOV3 and mouse 3LL cells by mouse *Cmah* gene transfection, resulting in successful antibody-dependent cell-mediated cytotoxicity (ADCC) against both types of target cells.

Furthermore, the 14F7hT antibody exhibited an anti-metastatic effect in C57BL/6 mice and inhibited tumor growth in BALB/c *nu/nu* mice implanted with these *Cmah*-transfected mouse and human cell lines, respectively. The latter result is the first demonstration of the antitumor effect of 14F7hT against a human cancer cell line. In summary, by using novel transgenic cell lines (3LL-*Cmah* and SKOV3-*Cmah*) we provide here further evidence of the therapeutic potential of the 14F7hT antibody against tumors of different origin expressing the GM3(Neu5Gc) neoantigen. This work also projects genetic *Cmah* transfection as a strategy for the preclinical evaluation of GM3(Neu5Gc)-targeting immunotherapies.

## Results

### Heterogeneous expression of GM3(Neu5Gc) and CMAH enzyme in different mouse cell lines

**Figure 1:**
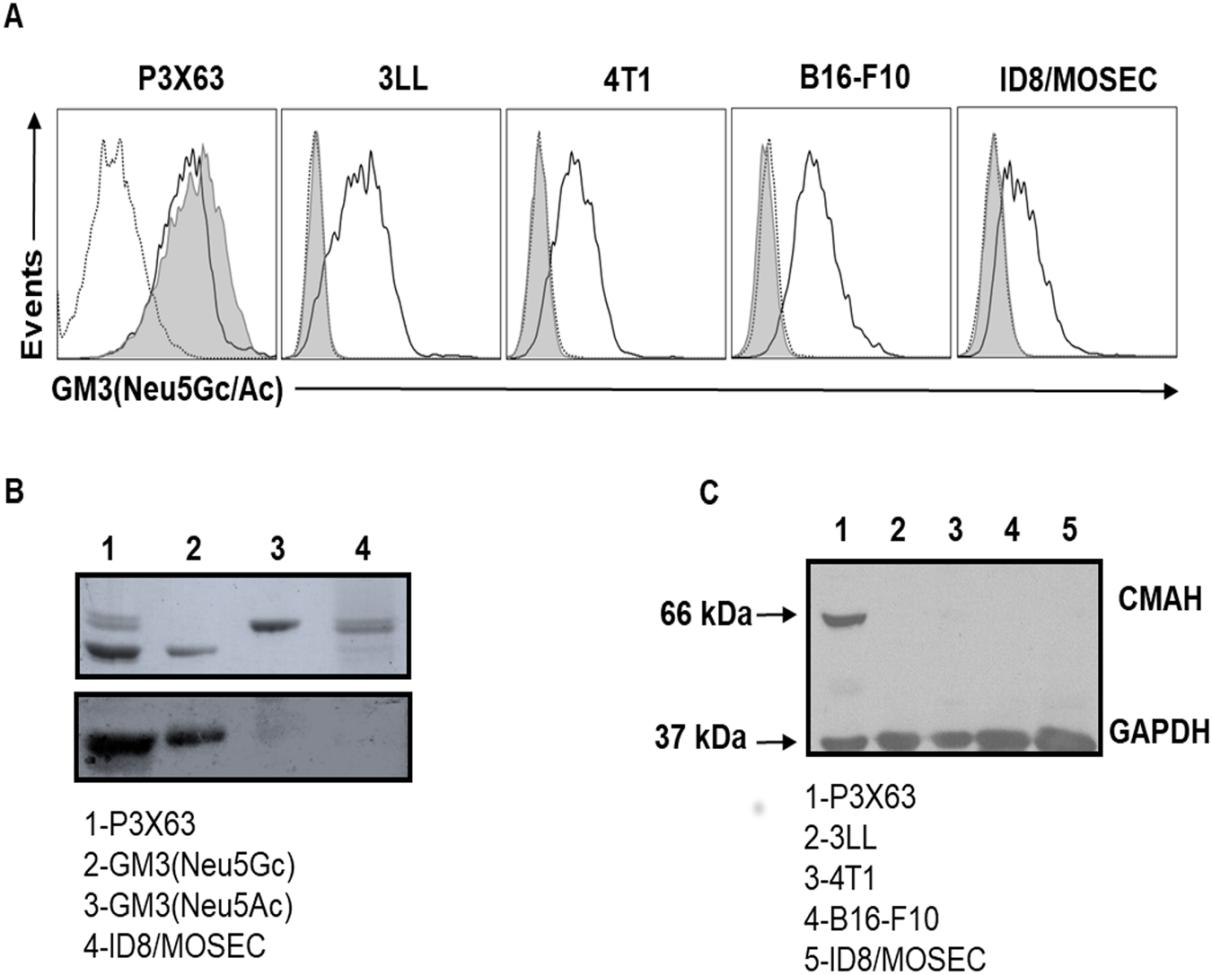
GM3(Neu5Gc) expression in mouse cell lines. **(A)** Mouse cell lines were stained with 10 µg/mL of 14F7hT (filled histogram), 7C1 (black line) or isotype-matched control antibody (itolizumab, dotted line) followed by a phycoerythrin(PE)-conjugated anti-human IgG + IgM antiserum. (**B)** GM3(Neu5Gc) content in ID8/MOSEC mouse ovarian epithelial cancer cells. Total lipids were extracted and monosialogangliosides were purified by ion exchange chromatography and visualized by orcinol staining (upper panel). For immunostaining, the plate was incubated with mouse 14F7 (10 μg/mL) and binding was revealed with an alkaline phosphatase-conjugated goat anti-mouse IgG antibody (bottom panel). Full-length blots are presented in Suppl. Fig. 1. (**C)** Expression of the mouse CMAH enzyme was determined in cell lysates by Western blot. Glyceraldehyde 3-phosphate dehydrogenase (GAPDH) was used as loading control. Full length blots are presented in Suppl. Fig. 2. Data are representative of three independent experiments.

The GM3(Neu5Gc) antigen, as recognized by the 14F7 antibody or its humanized variant, has previously been shown to be expressed on the P3X63 myeloma and L1210 lymphocytic leukemia mouse cell lines^22,30^. Given the abundant expression of Neu5Gc in murine cells, it would be expected that GM3(Neu5Gc) is generally expressed in mouse cell lines. However, while by flow cytometric analysis the surface expression of GM3(Neu5Gc) on P3X63 cells was confirmed by staining with 14F7hT, no binding was detectable in other mouse cell lines, including 3LL Lewis lung carcinoma, 4T1 mammary carcinoma, B16-F10 melanoma, and ID8/MOSEC ovarian cancer cell lines (Fig. 1A). In contrast, high intensity signals were detected for all cell lines following staining with 7C1, an engineered version of 14F7hT that recognizes both GM3(Neu5Gc) and GM3(Neu5Ac) gangliosides^29,35^, thus indicating GM3(Neu5Ac) expression in the 14F7hT non-binding cell lines. Indeed, high-performance thin layer chromatography (HPTLC) followed by chemical staining of monosialogangliosides by orcinol (Fig. 1B; upper panel) and immunostaining with 14F7 (Fig. 1B; lower panel), confirmed the predominant expression of GM3(Neu5Gc) in P3X63 cells, while as opposed, ID8/MOSEC cells expressed GM3(Neu5Ac).

Given the unexpectedly low surface expression of GM3(Neu5Gc) in 3LL, 4T1, B16-F10 and ID8/MOSEC cells, we investigated the expression of CMAH, the enzyme responsible for the generation of CMP-Neu5Gc by catalyzing the transfer of a hydroxyl group to CMP-Neu5Ac. Immunoblotting experiments revealed high CMAH expression levels in P3X63 cells, but only low or undetectable amounts of the enzyme in 3LL, 4T1, B16-F10 and ID8/MOSEC cells (Fig. 1C).

### GM3(Neu5Gc) surface expression on human cell lines

**Figure 2:**
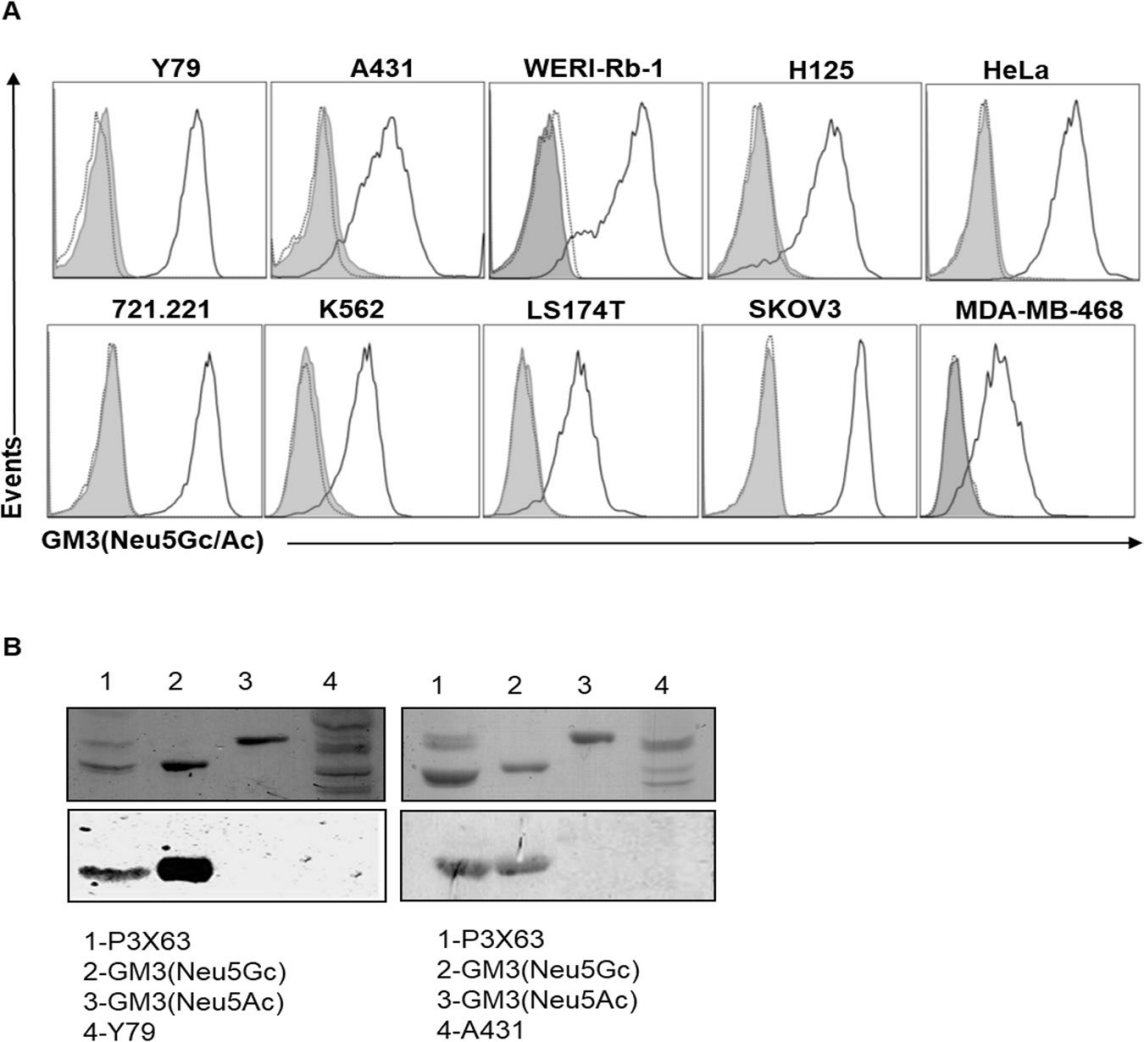
GM3(Neu5Gc) expression in human cell lines. (**A**) Human cell lines were stained with 10 µg/mL of 14F7hT (filled histogram), 7C1 (black line) or isotype-matched control antibody (itolizumab, dotted line) followed by a PE-conjugated anti-human IgG + IgM antiserum. (**B**) GM3 content in Y79 and A431 cell lines. Total lipids were extracted and monosialogangliosides were purified by ion exchange chromatography and visualized by orcinol staining (upper panel). For immunostaining, the plate was incubated with mouse 14F7 (10 μg/mL) and binding was revealed with an alkaline phosphatase-conjugated goat anti-mouse IgG antibody (bottom panel). Full-length blots are presented in Suppl. Fig. 1. Data are representative of three independent experiments.

Given that human cells lack a functional CMAH enzyme^4,5^, human cell lines are not expected to constitutively express the xenogeneic GM3(Neu5Gc) ganglioside. Nevertheless, few reports exist that mention the possibility that GM3(Neu5Gc)-targeting antibodies bind human cell lines, such as to Y79 and WERI-Rb-1 retinoblastoma^26,31,32^ or A431 epidermoid carcinoma cells^33^. Here, quantitative flow cytometry was employed for the screening of several well characterized human cell lines, including the Y79, WERI-Rb-1 and A431 cells. In no human cell line evidence for GM3(Neu5Gc) ganglioside surface expression was found using the 14F7hT antibody, neither on the latter, nor on H125 lung cancer, HeLa cervical cancer, 721.221 B lymphoblastoid, K562 myelogenous leukemia, SKOV3 ovarian cancer, MDA-MB-468 mamary carcinoma, and colorectal cell lines, LS174T, HCT 116, HT29, SW620 and SW1116 (Fig. 2A and Suppl. Fig. 3). In contrast, significant surface staining of all cell lines was observed using the 7C1 antibody, which binds to both GM3(Neu5Gc) and GM3(Neu5Ac). These findings indicate that GM3 gangliosides in the investigated human cell lines possess a Neu5Ac sialic acid moiety. Next, we confirmed these results using HPTLC followed by orcinol staining (Fig. 2B; upper panel) and immunostaining with 14F7 (Fig. 2B; lower panel), whereby GM3(Neu5Gc)-positive mouse P3X63 cells served as control. In these assays, GM3(Neu5Gc) was detected in P3X63 cells as expected, but not in the purified monosialoganglioside fractions from human Y79 and A431 cells.

### Hypoxia drives GM3(Neu5Gc) expression

**Figure 3:**
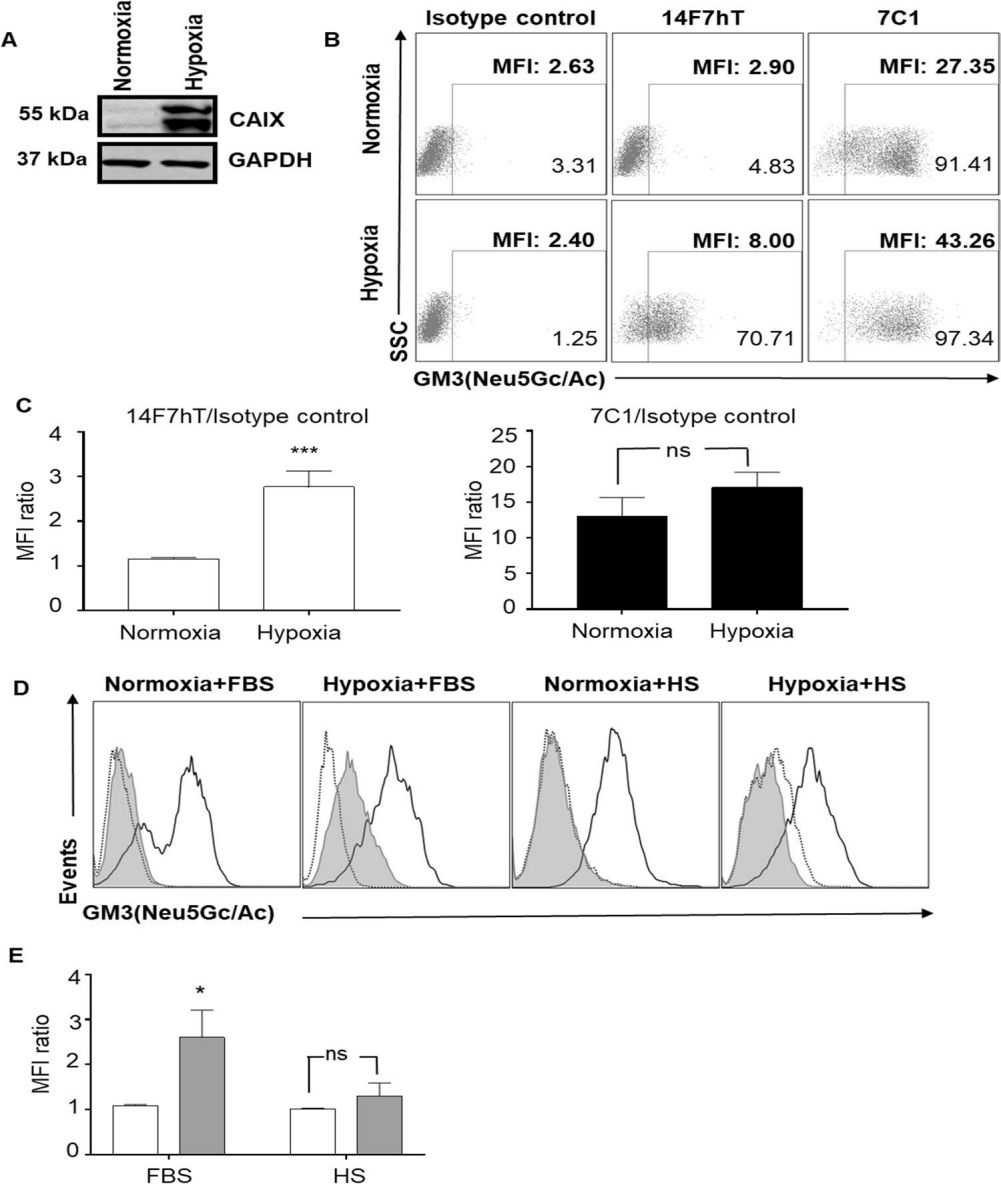
Expression of GM3(Neu5Gc) under hypoxic conditions on the SKOV3 human ovarian carcinoma cell line. SKOV3 cells were cultured under normoxic and hypoxic conditions with fetal bovine serum (FBS) for 7 days. (**A**) Expression of carbonic anhydrase IX (CAIX) as assessed by Western blot in cell lysates from SKOV3 cultures under normoxic or hypoxic conditions. GAPDH was used as loading control. Full-length blots are presented in Suppl. Fig. 2. (**B**) Cells were stained with 10 µg/mL of 14F7hT, 7C1 or isotype-matched control (itolizumab) followed by a fluorescein isothiocyanate (FITC)-conjugated anti-human IgG F(ab’)_2_. Numbers represent percentage of positive cells and mean fluorescence intensity (MFI) value of 14F7hT staining. (**C**) Quantitative analysis of the expression of GM3(Neu5Gc) (left panel) or GM3(Neu5Gc/Ac) (right panel) under hypoxic conditions (n = 7). Statistical analysis was performed using Student’s *t* test (*p* = 0.0006). (**D**) SKOV3 cells were cultured under normoxic or hypoxic conditions with FBS or HS for seven days. Cells were stained with 10 µg/mL of 14F7hT (filled histogram), 7C1 (black line) and isotype control (dotted line) followed by an FITC-conjugated anti-human IgG F(ab’)_2_. (**E**) Quantitative analysis of four independent experiments. Normoxia: white bar; hypoxia: grey bar. Statistical analysis was performed using Mann-Whitney *U* test.

Although a recently published hypothesis suggested *de novo* synthesis of Neu5Gc in human hypoxic tumor cells^12^, the most accepted explanation for the presence of Neu5Gc in human tumors is the incorporation of this sialic acid from exogenous sources^9–11^. Some studies have demonstrated that FBS is a rich source of Neu5Gc^10,25^. However, our results show that standard culture conditions may not guarantee Neu5Gc incorporation in GM3 gangliosides or compensate for the absence or reduction of the biosynthetic CMAH enzyme. Given that hypoxia has been reported to favor exogenous Neu5Gc incorporation by cancer cells^25^, we wondered if hypoxia might induce GM3(Neu5Gc) surface expression and allow for 14F7hT binding even in CMAH-inactive human cancer cells by metabolic incorporation of Neu5Gc-rich culture medium. To this end, the SKOV3 human ovarian cell line was cultured in FBS-containing medium under hypoxic conditions (1% O_2_) for seven days. Carbonic anhydrase IX (CAIX), a known marker of hypoxia was used to confirm this condition (Fig. 3A). As hypothesized, using flow cytometry we observed significant hypoxia-induced surface expression of GM3(Neu5Gc) in SKOV3 cells, as revealed by enhanced surface staining by 14F7hT (Fig. 3B,C). Fluorescence intensities of its engineered version 7C1, which also recognizes the constitutively expressed GM3(Neu5Ac) ganglioside, were high under normoxic conditions and further increased in hypoxia.

Recently, enhanced GM3(Neu5Gc) expression was measured by flow cytometry using 14F7 on human HeLa cells under hypoxic conditions, if cells were cultured with HS, but not with Neu5Gc-rich FBS^12^. The authors hypothesized that hypoxia induces *de novo* synthesis of Neu5Gc gangliosides by a CMAH domain substitute. In contrast, using 14F7hT we observed no hypoxia-induced expression of surface GM3(Neu5Gc) on human SKOV3 cells in the presence of HS (Fig. 3D,E). However, we observed hypoxia-induced GM3(Neu5Gc) expression following culture with Neu5Gc-rich FBS, supporting the concept of enhanced metabolic incorporation of Neu5Gc in human cancer cells under hypoxic conditions^25^.

### Recombinant expression of mouse CMAH enables GM3(Neu5Gc) expression in 3LL and SKOV3 cells

**Figure 4:**
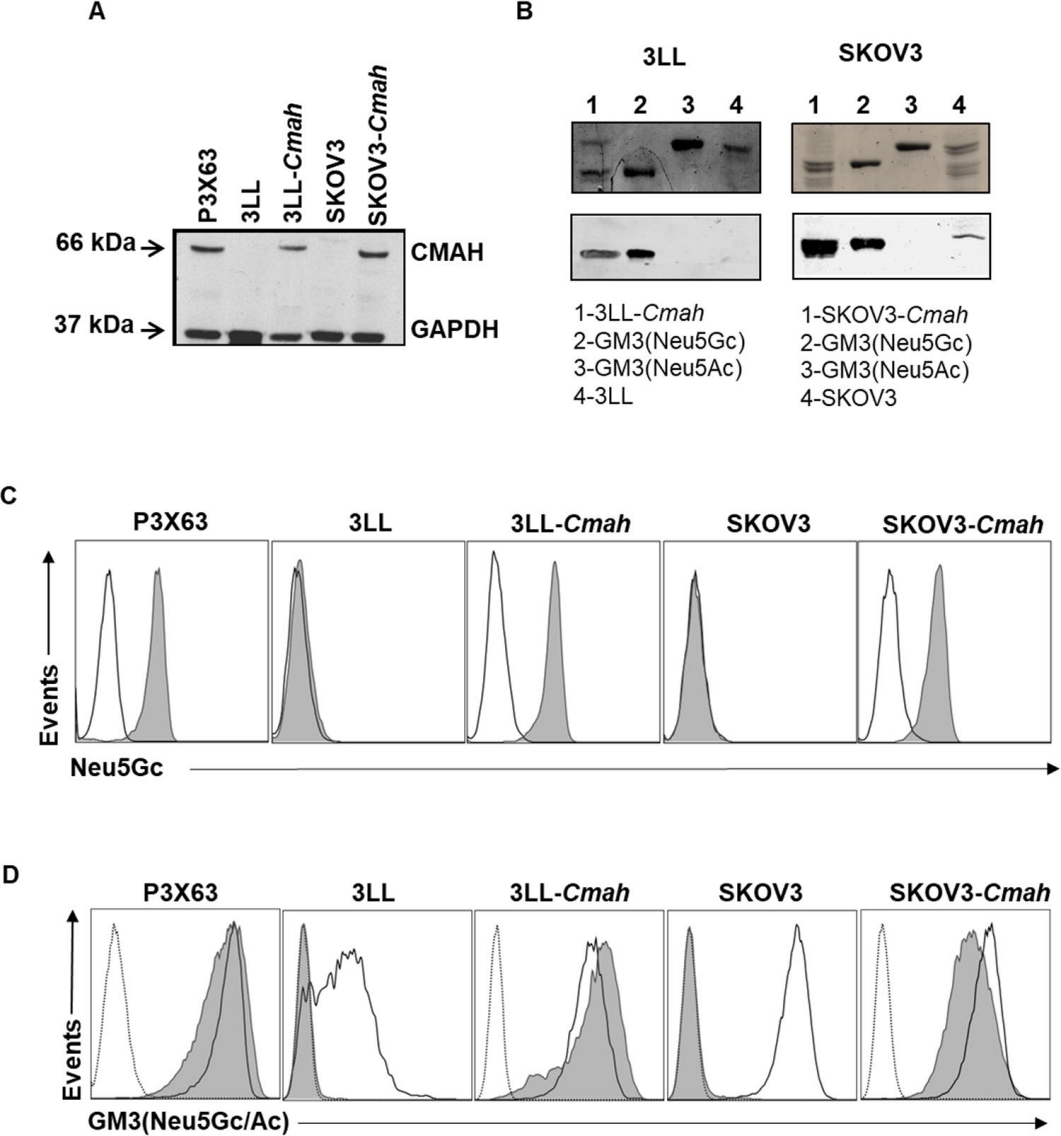
GM3(Neu5Gc) expression after transfection of the mouse *Cmah* gene in 3LL and SKOV3 cells. (**A**) Expression of the mouse CMAH enzyme as determined in cell lysates by Western Blot. GAPDH was used as loading control. Full-length blots are presented in Suppl. Fig. 2. (**B**) GM3(Neu5Gc) content in the transfected 3LL and SKOV3 cell lines. Total lipids were extracted and monosialogangliosides were purified by ion exchange chromatography and visualized by orcinol staining (upper panel). For immunostaining, the plates were incubated with mouse 14F7 and binding was revealed with an alkaline phosphatase-conjugated goat anti-mouse IgG antibody (bottom panel). Full-length blots are presented in Suppl. Fig. 1. (**C)** Total Neu5Gc expression in the surface of transfected and wild type 3LL and SKOV3 cell lines. A chicken anti-Neu5Gc antiserum (filled histogram) was used, followed by an FITC-conjugated goat anti-IgY antiserum. Chicken polyclonal IgY was used as negative control (black line). (**D)** 14F7hT binding to transfected and wild type 3LL and SKOV3 cell lines. Cells were stained with 10 µg/mL of 14F7hT (filled histogram), 7C1 (black line) or isotype-matched control antibody (itolizumab, dotted line) followed by a PE-conjugated anti-human IgG + IgM antiserum. P3X63 cells were used as positive control of GM3(Neu5Gc) expression. Data are representative of three independent experiments.

In order to obtain new models to evaluate the biological activity of 14F7hT beyond P3X63 and L1210 cells, we investigated if stable expression of GM3(Neu5Gc) can be reached in by mouse *Cmah* gene transfection. For this purpose, we selected the SKOV3 human cell line, with the inactive CMAH, as well as mouse 3LL cells, which exhibited undetectable expression of CMAH (see above). High levels of the recombinant CMAH protein were detected in transfected 3LL and SKOV3 cells, as assessed by immunoblotting (Fig. 4A). The enzyme was active, which was evidenced by the acquired capacity to generate detectable amounts of GM3(Neu5Gc), as revealed by purification of the monosialoganglioside fraction and subsequent chemical staining (Fig. 4B, upper panels) and immunostaining with 14F7 (Fig. 4B, bottom panels). Flow cytometric analysis using a chicken anti-Neu5Gc antiserum showed a general increase of surface Neu5Gc on transfected 3LL and SKOV3 cells (Fig. 4C). Moreover, significant GM3(Neu5Gc) surface expression was achieved in both 3LL-*Cmah* and SKOV3-*Cmah* cells, with acquisition of 14F7hT binding (Fig. 4D). The trace amounts of GM3(Neu5Gc) found in wild type SKOV3 by HPTLC in absence of detectable 14F7hT surface binding by flow cytometry can be attributed to intracellular incorporation of exogenous Neu5Gc from the FBS-containing culture medium.

### 14F7hT-mediated antitumor responses *in vitro* and *in vivo* in mouse *Cmah*-transfected cells

**Figure 5:**
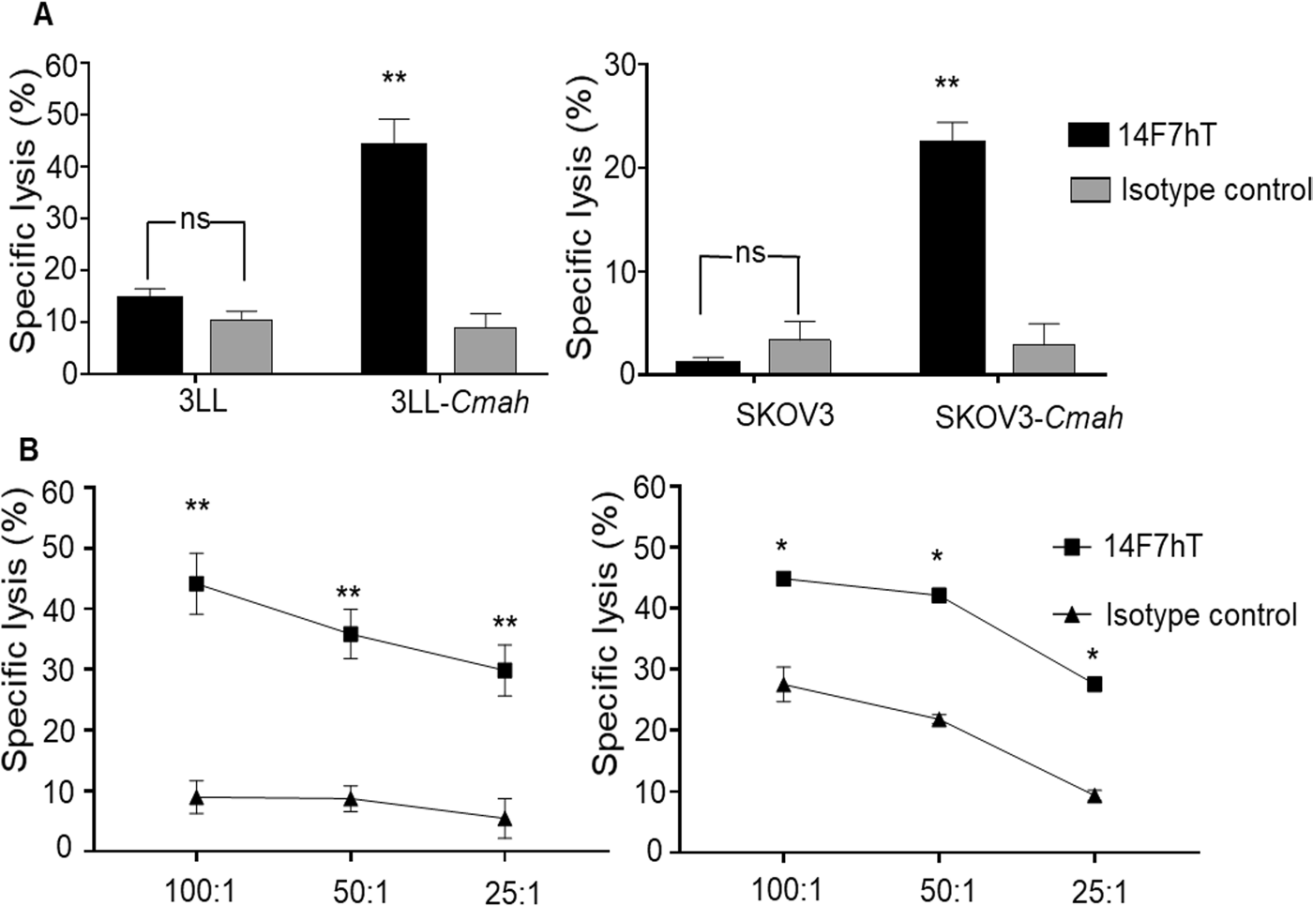
14F7hT-mediated antibody-dependent cell-mediated cytotoxicity of *Cmah*-transfected cell lines. **(A)** Induction of 14F7hT-mediated ADCC with human PBMC in mouse *Cmah*-transfected cell lines. Cytotoxicity was measured by a lactate dehydrogenase (LDH)-release assay. PBMC from human healthy donors were used as effector cells. (**B)** Cytotoxicity was evaluated at different effector:target cell ratios on 3LL-*Cmah* (left panel) and SKOV3-*Cmah* (right panel) cells. Isotype-matched antibodies (infliximab in left panels and rituximab in right panels) were used as negative controls. Statistical analysis was performed using Mann-Whitney *U* test. Data are representative of three independent experiments.

With the objective to assess the antitumor effect of 14F7hT, we evaluated the ability of this antibody to induce ADCC in the SKOV3-*Cmah* cell line. Human peripheral blood mononuclear cells (PBMCs) isolated from human healthy donors were used as effector cells (Fig. 5). The experiment was also performed using 3LL-*Cmah* as target cells **(**Fig. 5 and Suppl. Fig. 4). 14F7hT induced significant lysis only of mouse *Cmah*-transfected SKOV3 and 3LL cells, indicating efficient generation and surface localization of the GM3(Neu5Gc) antigen in these cells (Fig. 5A). 14F7hT-induced ADCC of the mouse *Cmah* gene-transfected target cells occurred in an effector:target cell (ET) ratio-dependent manner (Fig. 5B). Similar results were obtained when human natural killer (NK) cells were used as effector cells (Suppl. Fig. 5). Given the absence or low expression of GM3(Neu5Gc) on human and mouse cell lines, to date, the *in vivo* antitumor activity of 14F7hT had only been tested in a model using mouse P3X63 myeloma cells^23^. Therefore, we went on to test if 14F7hT mediated antitumor responses also *in vivo* against *Cmah*-transfected cells.

**Figure 6:**
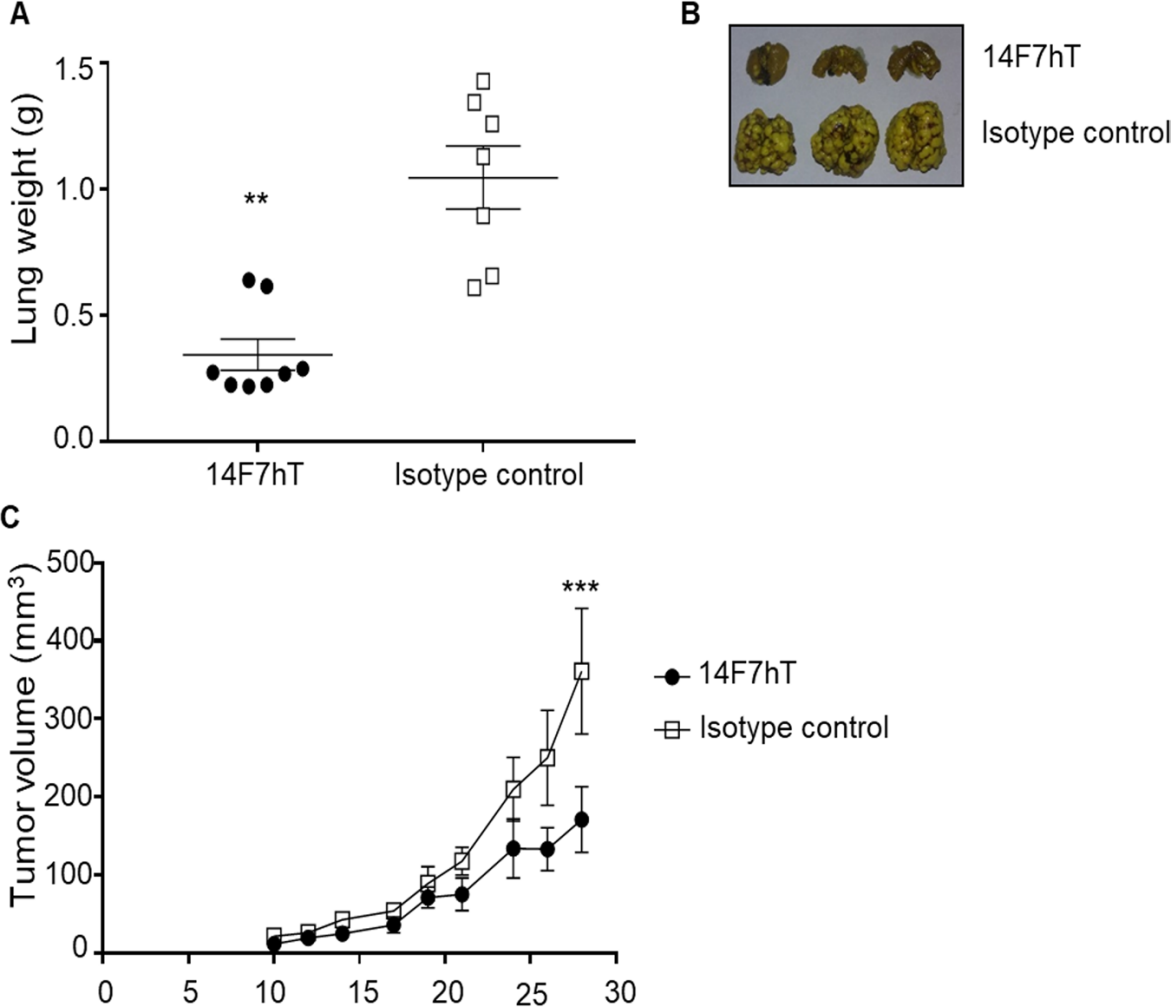
Antitumor effects of 14F7hT against *Cmah*-transfected cell lines *in vivo*. (**A,B)** Anti-metastatic effect of 14F7hT in the 3LL-*Cmah* model. Cells (0.25 × 10^6^) were injected into the lateral tail vein of C57BL/6 mice (n = 7–8) and 14FhT was administered intravenously in six doses until day 13. Weight (**A**) and representative macroscopic pictures (**B**) of the lungs upon euthanasia at day 21. Statistical analysis was performed using Mann-Whitney *U* test (*p* = 0.0012). (**C)** Antitumor effect of 14F7hT against SKOV3-*Cmah* cells. Cells (1 × 10^6^) were subcutaneously inoculated to BALB/c *nu/nu* mice and 14F7hT administered intraperitoneally three times a week for three weeks. Tumor diameters were measured with a caliper and tumor volume was calculated. Data represent mean ± standard error of the mean, n = 6–7. Statistical analysis was performed using the two-way ANOVA test with Sidak’s post-test (*p* = 0.0004). An isotype-matched antibody (itolizumab) was used as negative control. Data are representative of two independent experiments.

To this end, we first evaluated an experimental metastasis model based on 3LL cells. C57BL/6 mice were injected into the lateral tail vein with 3LL-*Cmah* cells (0.25 × 10^6^) and 14FhT was administered intravenously (0.5 mg/animal) in six doses from the following day to day 13. The metastatic burden was determined eight days later by weighting the lungs of the euthanized animals. 14F7hT-treated mice exhibited significantly lower (Mann-Whitney *U* test, *p* = 0.0012) and reduced size of the lung (Fig. 6A,B). In another model, BALB/c *nu/nu* mice were implanted subcutaneously with SKOV3-*Cmah* cells (1 × 10^6^) and 14F7hT was administered intraperitoneally (1 mg/animal) three times a week for three weeks, starting three days after implantation. As shown in Fig. 6C, a reduction of tumor growth was observed in 14F7hT-treated mice, which reached statistical significance at day 28 (two-way ANOVA, Sidak’s test, *p* = 0.0004). This result demonstrates for the first time, an *in vivo* antitumor effect of 14F7hT against human cancer cells expressing GM3(Neu5Gc).

## Discussion

In humans, Neu5Gc-sialoconjugates are found only in trace amounts in tissues^8^, due to the inactivity of the CMAH enzyme resulting from an exon deletion in the *Cmah* gene^4,5^. Although alternate pathways for Neu5Gc expression have been proposed in humans^12^, studies using *Cmah*−/− mice did not support such hypothesis, but instead highlighted a central role of dietary uptake and metabolic incorporation^7,36,37^. Indeed, by quantitative flow cytometry, we found no evidence for surface expression of the GM3(Neu5Gc) ganglioside in human cell lines. Furthermore, using HPTLC-immunostaining no binding of the 14F7 antibody was detected in the monosialoganglioside fractions isolated from Y79 and A431 cells. These data suggest that at least for GM3(Neu5Gc), putative alternate pathways cannot compensate for the human-specific loss of functional CMAH. The faint TLC-immunostaining signal intensities observed in extracts from SKOV3 cells most likely result from metabolic incorporation from FBS, yet cell surface levels of GM3(Neu5Gc) were not detectable by 14F7hT staining in flow cytometric analysis. It remains to be clarified whether the reported positive staining of 14F7 in human Y79 and WERI-Rb-1 cells^26^, in a non-commercial melanoma cell line^27^ and in the T24 bladder cancer cell line^28^, is predominantly related to labeling of intracellular antigens or to the metabolic incorporation of Neu5Gc from culture medium.

In contrast to human cells, CMAH is functional in mice, and thus responsible for the presence of Neu5Gc in tissues and cell lines^7^. The MB49 and MB49-I mouse bladder cancer cell lines were very recently reported to be stained by the mouse 14F7 antibody^28^. However, we observed only low or absent CMAH protein expression in the  murine cell lines that we tested, with the exception of the P3X63 cells.

 Indeed, previous studies using RT-PCR showed no transcription of the *Cmah* gene in mouse B16-F0 and F3II cell lines^24,34^. In mouse cell lines with reduced CMAH expression, no detectable surface expression of GM3(Neu5Gc) was found, indicating the lack of alternate biosynthetic pathways for the generation of this ganglioside. Notably, the parental 14F7 antibody has been well characterized by several methodological approaches, including crystallography^38^, glycan array technology^12^, and immunohistochemistry on human healthy and malignant tissue specimens^15^, while less is known about the recently described mouse IgG3 antibody Mab-1^33^. This antibody was reported to stain ID8/MOSEC cells by flow cytometry^33^, for which we detected neither relevant CMAH expression nor 14F7hT binding. The reported binding of Mab-1, in the absence of the GM3(Neu5Gc) biosynthetic CMAH pathway in ID8/MOSEC cells, warrants further investigations on this antibody, or in general, considerations on the use of culture media (potential exogenous source of Neu5Gc) for the assessment of Neu5Gc-targeting antibodies.

Proposed mechanisms for uptake and metabolic incorporation of Neu5Gc in human cells include fluid pinocytosis, lysosomal sialidase to release free Neu5Gc, and sialic acid transporter molecules^10,25^. Hypoxia has been shown to induce the transcription of sialin, a sialic acid transporter, in human Caco-2 and ZR-75-1 cancer cells and by this means to facilitate the cellular incorporation of sialic acids from culture medium *in vitro*^25^. Here, we found that hypoxia induced the surface expression of GM3(Neu5Gc) and 14F7hT binding in SKOV3 cells. Hypoxia-induced upregulation of GM3(Neu5Gc) surface expression was exclusively observed upon culture with Neu5Gc-rich FBS, but not with Neu5Gc-deficient HS, indicating the requirement of metabolic incorporation in absence of functional CMAH biosynthetic pathways. Conversely, in a recent study, hypoxic culture in Neu5Gc-deficient chicken serum and HS but not in Neu5Gc-rich FBS, enhanced the surface expression of GM3(Neu5Gc) on HeLa cells, and the authors suggested that CMAH-independent pathways may be induced in hypoxia^12^. According to the authors, their findings would indicate *de novo* synthesis of the sialic acid Neu5Gc. However, it remained largely unexplained why this alleged rescue of the CMAH activity did not occur in the presence of FBS.

Based on these findings, we decided to generate new tumor models to evaluate the biological activity of 14F7hT. Both, the CMAH-low mouse cell line 3LL and the human SKOV3 cells could be stably transfected to express the mouse CMAH enzyme, resulting in cell surface expression of Neu5Gc and Neu5Gc-containing GM3 ganglioside. Strong binding of 14F7hT to the surface of transfected, but not wild-type, 3LL and SKOV3 cells was confirmed by flow cytometric analysis. More importantly, 14F7hT was able to induce ADCC either with human PBMC or purified human NK cells in both mouse 3LL and human SKOV3 cells transfected with the mouse *Cmah* gene, while no cytotoxicity was observed against their wild-type counterparts. Moreover, the antibody exhibited antitumor effects *in vivo* in both models, which constitutes the demonstration that 14F7hT can target and mediate the killing of a non-hematological cancer cells expressing GM3(Neu5Gc). In the particular case of transfected SKOV3 cells, it is also the first evidence of the effect of 14F7hT on human cancer cells. We are currently conducting experiments to determine the 14F7hT-mediated mechanisms that can be relevant *in vivo*, including the participation of the cellular immunity.

Despite bearing a human IgG1 Fc, we do not rule out completely an ADCC effect. The interaction of human antibodies with mouse Fc receptors has been reported^39,40^. Furthermore, the antitumor effects of Rituxan and Herceptin were absent in FcRγ−/− nude mice^41^.

The present work highlights the importance of the GM3(Neu5Gc) ganglioside as a tumor-specific antigen and reinforces the hypothesis of exogenous Neu5Gc uptake and its enhanced expression under hypoxic conditions, as an explanation for its presence in human malignant tissues. More importantly, our results pave the way to a deeper understanding of the antitumor mechanisms of 14F7hT, which emerges as a significant immunotherapeutic drug against tumors of different histological origins expressing this neoantigen. Furthermore, antitumor effects of 14F7hT against *Cmah-*transfected, GM3(Neu5Gc)-expressing human and mouse cancer cells could be demonstrated in *in vivo* models, promoting *Cmah* gene transfection as a reliable strategy for pre-clinical evaluation of GM3(Neu5Gc)-targeting therapeutic drugs.

## Materials and Methods

### Cells

Mouse P3X63-Ag8.653 (P3X63, non-immunoglobulin-producing myeloma, CRL-8375), B16-F10 (melanoma, CRL-6475), 4T1 (breast carcinoma, CRL-2539), human SKOV3 (ovarian adenocarcinoma, HTB-77), K562 (chronic myeloid leukemia, CCL-243), HeLa (cervix adenocarcinoma, CCL-2), A431 (epidermoid carcinoma, CRL-1555), MDA-MB-468 (mammary carcinoma, HTB-132), WERI-Rb-1 (retinoblastoma, HTB-169) and Y79 (retinoblastoma, HTB-18) and colorectal cell lines LS174T (CL-188), HCT116 (CCL-247), HT29 (HTB-38), SW620 (CCL-227) and SW116 (CCL-233) were purchased from the American Type Culture Collection (ATCC, Rockville, MD). The highly metastatic D122 clone derived from mouse 3LL Lewis lung carcinoma (here referred to as 3LL cells)^42^ and mouse ID8/MOSEC from an ovarian epithelial cell line^43^, as well as human H125 derived from lung cancer^44^ and 721.221 from a lymphoblastoid B cell line^45^, were also used. Cells were cultured in Dulbecco’s modified Eagles medium (DMEM) or RPMI-1640 from Gibco-BRL (Paisley, UK), supplemented with 10% FBS (PAA, Pasching, Austria) or HS (Sigma-Aldrich, St. Louis, MO) and 1% of Penicillin/Streptomycin. For hypoxia experiments cells were cultured in an InVivo2 400 incubator (Ruskinn Technology, Glamorgan, UK) at 1% O_2_ for seven days.

Blood from healthy donors was collected upon written informed consent in accordance with the guidelines of, and approved by the ethical committees of the Center of Molecular Immunology, Havana, Cuba, and Canton Bern, Switzerland. PBMC from human healthy donors were isolated by density gradient centrifugation using Ficoll-Paque^TM^ PLUS (GE-Healthcare, Uppsala, Sweden) and NK cells were purified using the NK cell negative selection kit from Stem Cell Technologies (Cambridge, UK), according to the manufacturer’s instructions. These cells were collected upon informed consent and in compliance with the protocols approved by CIM. PBMC and NKs were cultured in RPMI medium supplemented with FBS at 1 or 10%, at 37 °C and 5% CO_2._

### Antibodies

Humanized antibodies (IgG1, κ) 14F7hT, specific for the GM3(Neu5Gc) ganglioside^22^ and its engineered version 7C1, which binds to both the Neu5Ac and Neu5Gc variants of GM3^29^, and mouse 14F7 monoclonal antibody^21^, used for TLC-immunostaining, were purified from transfectoma by Protein A Affinity Chromatography (GE-Healthcare) and analyzed by SDS-PAGE under non-reducing conditions. A table the binding properties of these antibodies is included as supplementary information (Supplementary Table 1). Itolizumab (T1hT), specific for the human CD6 molecule^46^; rituximab (Rituxan; F. Hoffmann-La Roche, Basel, Switzerland), which recognizes the human CD20 molecule, and infliximab (Remicade, Merck/Schering Plough Pharmaceuticals LLC, North Wales, PA), specific for human TNF-α were used as isotype-matched controls.

### Animals

BALB/c *nu/nu* and C57BL/6 mice (female, 8–10 weeks-old) were purchased from the Center for Laboratory Animal Production (CENPALAB, Havana, Cuba). All procedures were performed in compliance with the protocols approved by the Institutional Committee for the Care and Use of Laboratory Animals of the CIM (CICUAL, 0017/2008). Animals were sacrificed by cervical dislocation, minimizing their suffering.

### Cloning of the mouse *Cmah* gene and selection of transfected cells

Total RNA from splenocytes of C57BL/6 mice was extracted with the Trizol reagent (Gibco-BRL). First strand cDNA synthesis and PCR amplification of the *Cmah* gene was performed using the Access RT-PCR kit, as described by the manufacturer (Promega, Madison, WI), with the following oligonucleotides:

5′-GATATCCACCATGATGGACAGGAAACAGACA-3′

5′-CTCGAGTCACAGTGCATTAGGAACGA-3′.

The purified PCR product was cloned into the pST-Blue-1 vector (Novagen, Darmstadt, Germany) for sequencing, digested *Eco*RV/*Xho*I and cloned into the equally digested pcDNA3 vector (Invitrogen, Paisley, UK). 3LL and SKOV3 cells were transfected with the *Pvu*I-linearized pcDNA3-*Cmah* vector using the lipofectamine LTX and Plus reagent (Invitrogen). Restriction and modification enzymes were purchased from New England Biolabs (Ipswich, MA). DMEM supplemented with 10% FBS and G418 at 1 mg/mL (Gibco-BRL) was used for the selection of transfected cells.

### Lipid extraction and thin layer chromatography-immunostaining

Cell lysates were homogenized and total lipids were extracted in chloroform:methanol:water solution (4:8:3, v:v:v). The extracts were centrifuged (1000 *g*, 15 min), the supernatant evaporated and the pellet dissolved in chloroform:methanol solution (1:1, v:v) for overnight incubation at 4 °C. After centrifugation, the dry samples were dissolved in chloroform:methanol:water (30:60:8,v:v:v) to be applied to a DEAE-Sephadex A-25 column (GE Healthcare). The acidic lipid fraction was eluted with 0.02 M sodium acetate in methanol. Thin layer chromatography (TLC) of gangliosides was performed as previously described^47^, with minor modifications. Briefly, HPTLC plates (Merck, Darmstadt, Germany) and chloroform:methanol:0.25% KCl in 2.5 M NH_3_ (5:4:1, v:v:v) as solvent were used for the glycolipid fractionation. Equal amounts of monosialogangliosides were applied and orcinol reactive was used for chemical staining. For immunostaining, plates were soaked for 75 s on hexane containing 0.1% polyisobutylmethacrylate (Sigma-Aldrich), dried for 1 h and then sealed with 1% polyisobutylmethacrylate solution. After drying and blocking with phosphate buffered saline-bovine seroalbumin (PBS-BSA 1%, w:v), plates were incubated with mouse 14F7 (10 μg/mL), washed with PBS, and an alkaline phosphatase-conjugated goat anti-mouse IgG + IgM (H + L) antiserum (Jackson Immunoresearch, West Grove, PA) was added. The reaction was developed with a substrate kit according to the manufacturer (Bio-Rad, Hercules, CA). Reaction was stopped by adding water. GM3(Neu5Gc) and GM3(Neu5Ac) used as positive controls were isolated from horse and dog erythrocytes, respectively^48^.

### Flow cytometry assays

Cells were incubated with 14F7hT, 7C1 or isotype-matched control antibodies (10 µg/mL) on ice for 30 min and washed with PBS-BSA 1%. Binding was detected by incubation with an FITC-conjugated rabbit anti-human IgG F(ab’)_2_ (Dako, Glostrup, Denmark) or a PE-conjugated goat anti-human IgG + IgM antiserum (Jackson Immunoresearch) for 30 min on ice.

For the detection of the total Neu5Gc-sialoconjugates, cells were incubated with a chicken anti-Neu5Gc antiserum (Biolegend, San Diego, CA) in binding buffer supplied by the manufacturer for 1 h on ice. After washing with cold PBS, binding was detected with an FITC-conjugated goat anti-chicken IgY antiserum (Biolegend). Chicken polyclonal IgY (Biolegend) was used as negative control.

MFI and percentage of stained cells were determined in a FACScalibur or a FACSVerse both from Becton Dickinson (Franklin Lakes, NJ) or a Gallios (Beckman Coulter, Brea, CA) cytometers. The FlowJo 10 (Tree Star, Ashland, OR) software was used for the analysis.

### Immunoblotting

Cell lysates were prepared in RIPA buffer (PBS, 1% Nonidet P-40, 0.5% sodium deoxycholate, 0.1% SDS) with 50 mM NaF, 1 mM Na_3_VO_4_, 5 mM EDTA, and 1 mM PMSF, all from Sigma-Aldrich that was freshly added to the lysis solution before each experiment. Similar quantities of lysates were applied to SDS-PAGE gels and transferred to polyvinylidine difluoride membranes (Millipore, Darmstadt, Germany). Membranes were blocked with Tris-buffered saline-Tween 20 0.1% (TBS-t) and 5% non-fat milk (Sigma-Aldrich) for 1 h at room temperature in agitation and incubated overnight with specific antibodies against the CMAH enzyme (Santa Cruz, Dallas, TX), CAIX (Novus Biologicals, Littleton, CO) and GAPDH (Cell Signaling; Danvers, MA). Binding was revealed with a horseradish peroxidase-conjugated anti-rabbit antiserum (Cell Signaling) followed by Luminata Forte (Millipore).

### Antibody-dependent cell-mediated cytotoxicity assay

ADCC was measured in an LDH-release assay. To this end, target cell lines were pre-incubated with the 14F7hT antibody for 30 min. Then, PBMC or NK, as effector cells, were added at different effector:target ratios in RPMI medium with 1% FBS. After 4 h incubation at 37 °C and 5% CO_2_, the supernatants were collected and the LDH activity was measured using a cytotoxicity detection kit (Roche, Mannheim, Germany), according to the manufacturer’s instructions. The absorbance of the product was measured at 490 nm with 630 nm filter in an ELISA reader ELX800 (DIALAB GmbH, Wiener Neudorf, Austria). Maximum release (high control) of LDH was determined in cells treated with 1% Triton X-100, while spontaneous release levels were measured in cells without antibody. Cells incubated with the antibodies (low control) and effector cells alone were included as controls. The percentage of specific lysis was calculated according to the following formula:

$${\rm{C}}{\rm{y}}{\rm{t}}{\rm{o}}{\rm{t}}{\rm{o}}{\rm{x}}{\rm{i}}{\rm{c}}{\rm{i}}{\rm{t}}{\rm{y}}({\rm{ \% }})=\frac{({\rm{e}}{\rm{f}}{\rm{f}}{\rm{e}}{\rm{c}}{\rm{t}}{\rm{o}}{\rm{r}}:{\rm{t}}{\rm{a}}{\rm{r}}{\rm{g}}{\rm{e}}{\rm{t}}\,{\rm{c}}{\rm{e}}{\rm{l}}{\rm{l}}\,{\rm{m}}{\rm{i}}{\rm{x}}-{\rm{e}}{\rm{f}}{\rm{f}}{\rm{e}}{\rm{c}}{\rm{t}}{\rm{o}}{\rm{r}}\,{\rm{c}}{\rm{e}}{\rm{l}}{\rm{l}}\,{\rm{c}}{\rm{o}}{\rm{n}}{\rm{t}}{\rm{r}}{\rm{o}}{\rm{l}})-{\rm{l}}{\rm{o}}{\rm{w}}\,{\rm{c}}{\rm{o}}{\rm{n}}{\rm{t}}{\rm{r}}{\rm{o}}{\rm{l}}}{{\rm{h}}{\rm{i}}{\rm{g}}{\rm{h}}\,{\rm{c}}{\rm{o}}{\rm{n}}{\rm{t}}{\rm{r}}{\rm{o}}{\rm{l}}-{\rm{l}}{\rm{o}}{\rm{w}}\,{\rm{c}}{\rm{o}}{\rm{n}}{\rm{t}}{\rm{r}}{\rm{o}}{\rm{l}}}\ast 100$$

where:

effector:target cell mix = LDH released by mix (antibody + effector cells + target cells); effector cell control = LDH released by effector cells; low control = LDH released by target cells incubated with the antibody without effector cells; high control = LDH released by target cells treated with Triton X-100 (Sigma-Aldrich) at 1%.

ADCC was also evaluated by flow cytometry. Target cells were stained with CFSE (1 µM) for 5 min. Then, FBS was added and cells were washed twice with PBS. After incubation with antibodies and effector cells (as above), specific lysis was measured by TO-PRO-3 (Invitrogen) staining in CFSE-labeled cells. The percentage of specific lysis was calculated according to the following formula:

$$\mathrm{Cytotoxicity}( \% )=\frac{{\rm{effector}}:{\rm{target}}\,{\rm{cell}}\,{\rm{mix}}-{\rm{low}}\,{\rm{control}}}{{\rm{high}}\,{\rm{control}}-{\rm{low}}\,{\rm{control}}}\ast 100$$

where:

effector:target cell mix = TO-PRO-3 staining in CFSE-labeled cells (target cells); low control = TO-PRO-3 staining in CFSE-labeled cells (target cells) incubated with the antibody without effector cells; high control = TO-PRO-3 staining in CFSE-labeled cells (target cells) treated with Triton X-100 (Sigma-Aldrich) at 1%.

### Antitumor assay

C57BL/6 mice were injected into the lateral tail vein with 3LL-*Cmah* cells (0.25 × 10^6^) and randomized. The 14FhT or isotype-matched control antibodies were administered (0.5 mg/animal) intravenously in six doses starting the following day and then every two-three days until day 13. At day 21, mice were euthanized and lungs were weighted as a measure of metastatic burden.

BALB/c *nu/nu* mice were implanted subcutaneously with SKOV3-*Cmah* cells (1 × 10^6^) and randomized. The 14F7hT or isotype-matched control antibodies were administered (1 mg/animal) intraperitoneally from day 3, three times a week for three weeks. Tumor diameters were measured every 2–3 days with a caliper and tumor volume was calculated according to the following formula:

$${\rm{T}}{\rm{u}}{\rm{m}}{\rm{o}}{\rm{r}}\,{\rm{v}}{\rm{o}}{\rm{l}}{\rm{u}}{\rm{m}}{\rm{e}}\,{({\rm{m}}{\rm{m}}}^{3})=\pi /6\times {\rm{m}}{\rm{a}}{\rm{j}}{\rm{o}}{\rm{r}}\,{\rm{d}}{\rm{i}}{\rm{a}}{\rm{m}}{\rm{e}}{\rm{t}}{\rm{e}}{\rm{r}}\times {({\rm{m}}{\rm{i}}{\rm{n}}{\rm{o}}{\rm{r}}{\rm{d}}{\rm{i}}{\rm{a}}{\rm{m}}{\rm{e}}{\rm{t}}{\rm{e}}{\rm{r}})}^{2}$$

### Statistical analyses

All statistical analyses were performed using GraphPad software version 7 (La Jolla, CA). For ADCC experiments and the anti-metastatic effect the non-parametric Mann-Whitney *U* test was used. The parametric Student’s *t* test was applied to analyze data from hypoxia culture. For comparison of tumor volume (factors: treatment group and day) a two-way ANOVA was performed followed by mean multiple comparison by Sidak’s test.

## Supplementary information

Supplementary figures
