# Supplementary material for: Antitumor effects of the GM3(Neu5Gc) ganglioside-specific humanized antibody 14F7hT against Cmah-transfected cancer cells
Source: Sci Rep. 2019 Jul 9;9:9921. doi: 10.1038/s41598-019-46148-1 (PMC6616363; doi:10.1038/s41598-019-46148-1)
Supplement: Supplementary file 1 — Supplementary figures [file 41598_2019_46148_MOESM1_ESM.pdf]

TITLE: Antitumor effects of the GM3(Neu5Gc) ganglioside-specific humanized antibody 14F7hT against *Cmah*-transfected cancer cells.

Running title: Targeting of GM3(Neu5Gc)-expressing *Cmah*-transfected cells by a humanized antibody.

AUTHORS: Denise Dorvignit[1], Kayluz F. Boligan[2], Ernesto Relova-Hernández[1], Marilyn Clavell[1], Armando López[3], Mayrel Labrada[1], Hans-Uwe Simon[2], Alejandro López-Requena[1$], Circe Mesa[1*#], Stephan von Gunten[2*]

ADDRESSES: [1]Immunobiology Division, Center of Molecular Immunology, Havana 11600, Cuba; [2]Institute of Pharmacology, University of Bern, Bern 3010, Switzerland; [3]Animal House Department, Center of Molecular Immunology, Havana 11600, Cuba.

[$] Current address: Ablynx, Technologiepark 21, 9052 Zwijnaarde, Belgium.

*contributed equally to this work

[#] Corresponding author:
Circe Mesa, PhD
Center of Molecular Immunology
P.O.Box 16040, Havana 11600,
Cuba.
Fax: (537)-2720644
Email: circe@cim.sld.cu

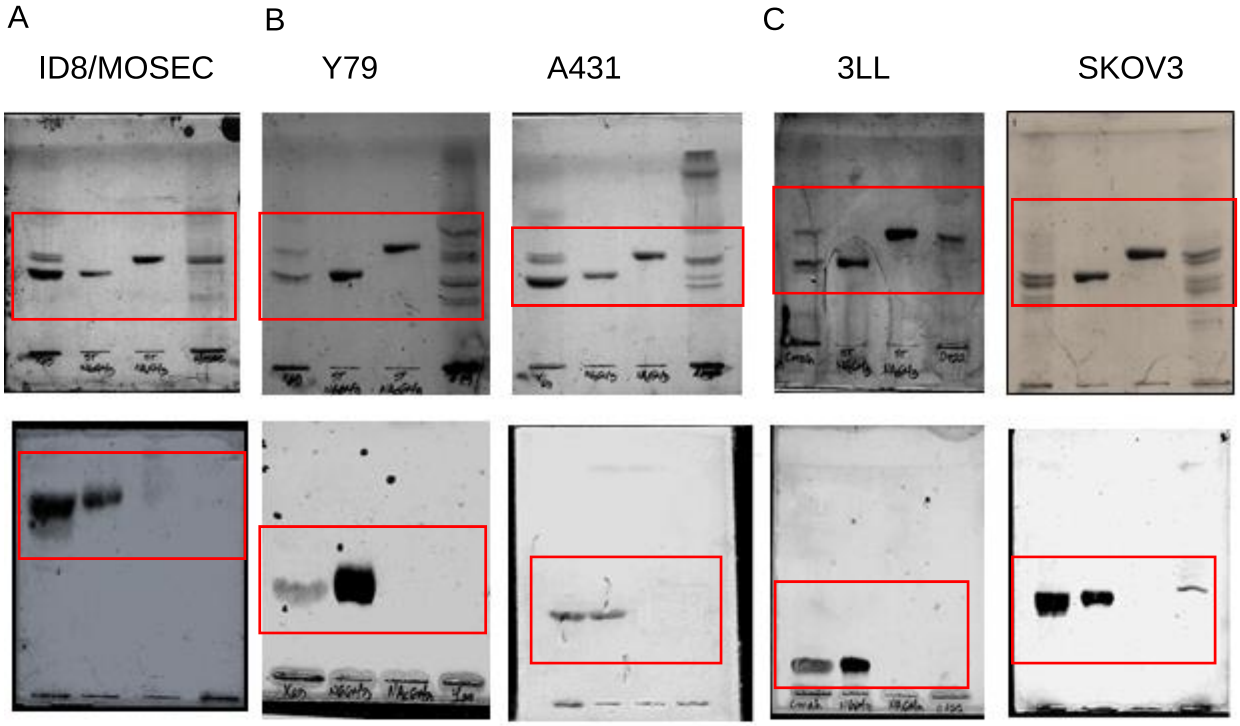

Supplementary figure 1. Original scans of HPTLC plates. Uncropped scans corresponding to images shown in Figure 1B (A), Figure 2B (B) and Figure 4B (C). Upper panels: orcinol staining; bottom panels: 14F7 immunostaining.

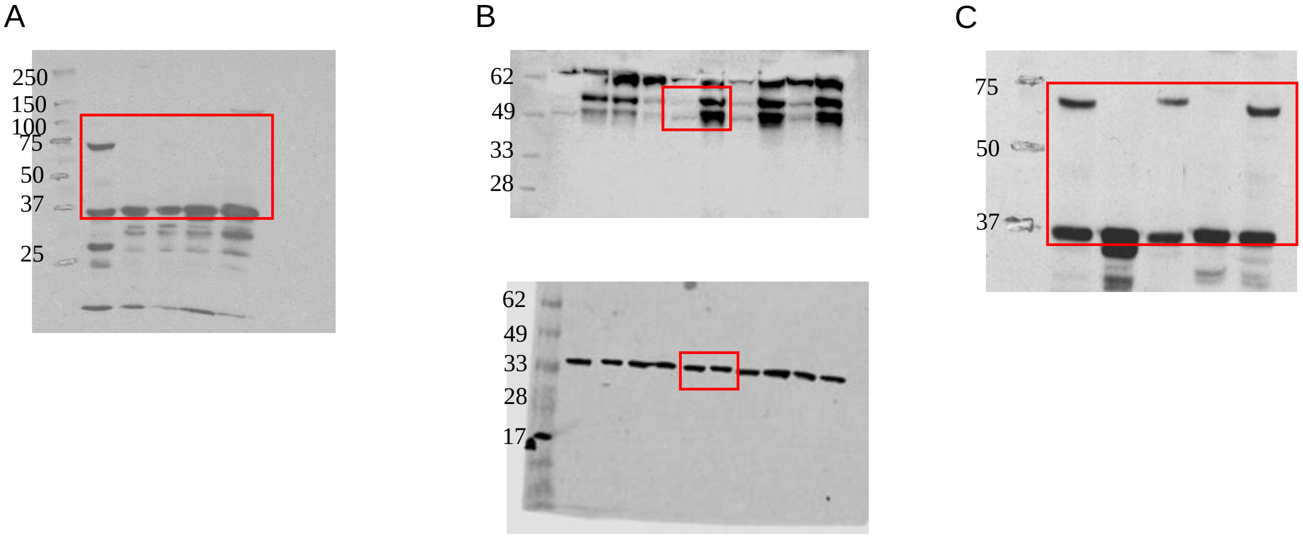

Supplementary figure 2. Original scans of Western blots. Uncropped scans corresponding to images shown in Figure 1C (A), Figure 3A (B) and Figure 4A (C).

Supplementary Figure 2

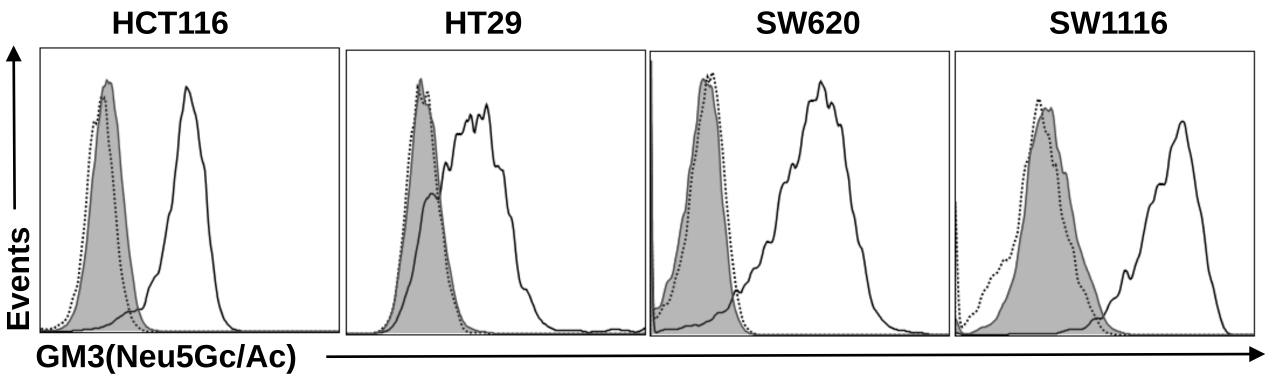

Supplementary figure 3. GM3(Neu5Gc) expression in human colon cancer cell lines. Cells were stained with 10 µg/mL of 14F7hT (filled histogram), 7C1 (black line) or isotype-matched control antibody (itolizumab, dotted line) followed by a PE-conjugated anti-human IgG+IgM antiserum. Data are representative of two independent experiments.

Supplementary Figure 3

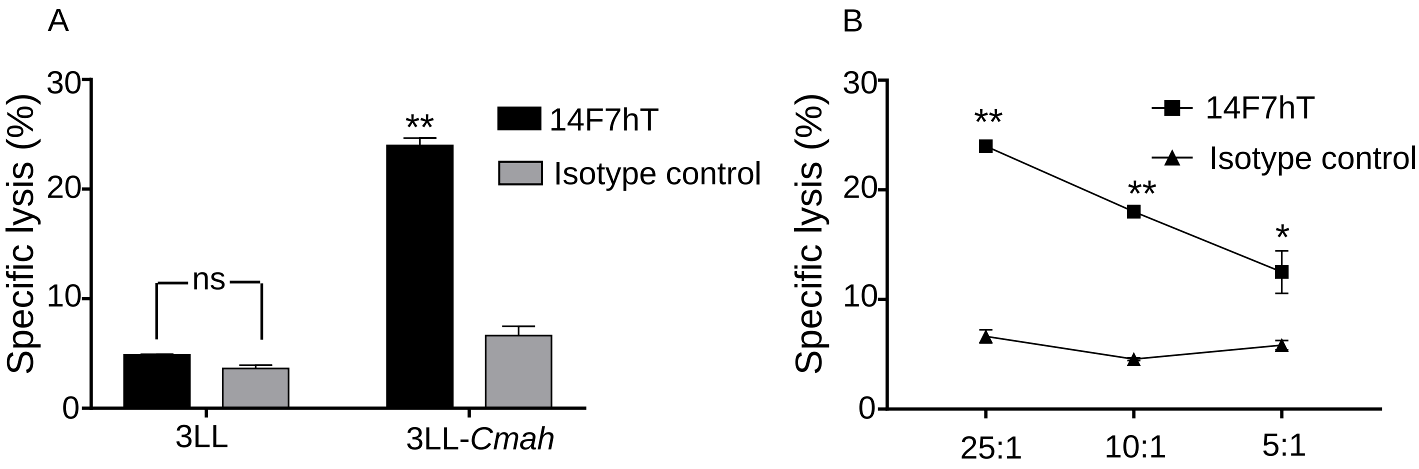

Supplementary figure 4. 14F7hT-induced antibody-dependent cell-mediated cytotoxicity in recombinant CMAH-expressing cell lines. A) Induction of antibody-dependent cell-mediated cytotoxicity (ADCC) by 14F7hT with human PBMC in the mouse *Cmah*-transfected cell line 3LL-*Cmah*. Cells were stained with CFSE and cytotoxicity was measured using TO-PRO-3 dye by flow cytometry assay. PBMC from human healthy donors were used as effector cells. B) Cytotoxicity was evaluated at different effector:target cell ratios. Isotype-matched antibody (infliximab) was used as negative control. Statistical analysis was performed using Mann-Whitney *U* test. Data are representative of two independent experiments.

A

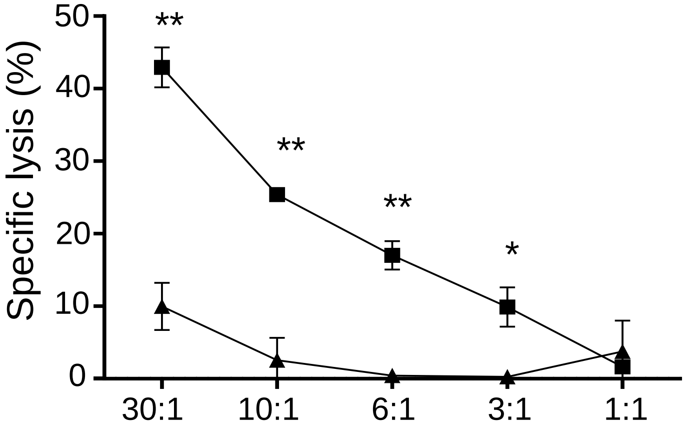

Supplementary figure 5. 14F7hT-opsonized 3LL-*Cmah* cells induce NK cells activation. A) Induction of antibody-dependent cell-mediated cytotoxicity (ADCC) by 14F7hT in the mouse *Cmah*-transfected cell line 3LL-*Cmah*. Cytotoxicity was measured by an LDH-release assay. NK cells from human healthy donors were used as effector cells. Cytotoxicity was evaluated at different effector:target cell ratios. Isotype-matched antibody (infliximab) was used as negative control. Statistical analysis was performed using Mann-Whitney *U* test. Data are representative of three independent experiments.

Supplementary table 1: Anti-ganglioside antibodies used in this study

Antibody	Isotype	Origin	Specificity	Antigen source	Assays	Affinity	Structural data	Selected references
14F7	mouse IgG1, κ	immunized BALB/c mouse	GM3(Neu5Gc)	mouse, horse, human[a], synthetic	ELISA, HPTLC, FACS, IHC, RIS	2-25 nM[b]	Fab, scFv[c]	1-9
14F7hT	human IgG1, κ	14F7 humanized by disruption of predicted T cell epitopes	GM3(Neu5Gc)	mouse, horse, human[a]	ELISA, HPTLC, FACS, IHC	ND	ND	10-12
7C1	human IgG1, κ	14F7hT binding site engineered for extended specificity	GM3(Neu5Gc)	mouse, horse	ELISA, HPTLC, FACS	ND	ND	11, 13
			GM3(Neu5Ac)	mouse, dog, human	ELISA, HPTLC, FACS			

[a]Tumor tissues.

[b]Measured by non-competitive ELISA.

[c]14F7 VH with alternative VL.

HPTLC: high-performance thin layer chromatography; IHC: immunohistochemistry; RIS: radioimmunoscintigraphy; ND: not determined.

References

1. Carr, A. *et al.* A mouse IgG1 monoclonal antibody specific for N-glycolyl GM3 ganglioside recognized breast and melanoma tumors. *Hybridoma* **19**, 241-247, https://doi.org/10.1089/02724570050109639 (2000).
2. Krengel, U. *et al.* Structure and molecular interactions of a unique antitumor antibody specific for N-glycolyl GM3. *J Biol Chem* **279**, 5597-5603, https://doi.org/10.1074/jbc.M311693200 (2004).
3. Rojas, G. *et al.* Light-chain shuffling results in successful phage display selection of functional prokaryotic-expressed antibody fragments to N-glycolyl GM3 ganglioside. *J Immunol Methods* **293**, 71-83, https://doi:10.1016/j.jim.2004.07.002 (2004).
4. Oliva, J. P. *et al.* Clinical evidences of GM3 (NeuGc) ganglioside expression in human breast cancer using the 14F7 monoclonal antibody labelled with (99m)Tc. *Breast Cancer Res Treat* **96**, 115-121, https://doi.org/10.1007/s10549-005-9064-0 (2006).
5. Roque-Navarro, L. *et al.* Anti-ganglioside antibody-induced tumor cell death by loss of membrane integrity. *Mol Cancer Ther* **7**, 2033-2041, https://doi.org/10.1158/1535-7163.MCT-08-0222 (2008).
6. Labrada, M. *et al.* Direct validation of NGcGM3 ganglioside as a new target for cancer immunotherapy. *Expert Opin Biol Ther* **10**, 153-162, https://doi:10.1517/14712590903443084 (2010).
7. Lahera, T. *et al.* Prognostic Role of 14F7 Mab Immunoreactivity against N-Glycolyl GM3 Ganglioside in Colon Cancer. *J Oncol* **2014**, 482301, https://doi.org/10.1155/2014/482301 (2014).
8. Blanco, R. *et al.* Prognostic Significance of N-Glycolyl GM3 Ganglioside Expression in Non-Small Cell Lung Carcinoma Patients: New Evidences. *Patholog Res Int* **2015**, 132326, https://doi.org/10.1155/2015/132326 (2015).
9. Bjerregaard-Andersen, K. *et al.* Crystal structure of an L chain optimised 14F7 anti-ganglioside Fv suggests a unique tumour-specificity through an unusual H-chain CDR3 architecture. *Sci Rep* **8**, 10836, https://doi:10.1038/s41598-018-28918-5 (2018).
10. Fernandez-Marrero, Y. *et al.* A cytotoxic humanized anti-ganglioside antibody produced in a murine cell line defective of N-glycolylated-glycoconjugates. *Immunobiology* **216**, 1239-1247, https://doi.org/10.1016/j.imbio.2011.07.004 (2011).
11. Casadesus, A. V. *et al.* A shift from N-glycolyl- to N-acetyl-sialic acid in the GM3 ganglioside impairs tumor development in mouse lymphocytic leukemia cells. *Glycoconj J* **30**, 687-699, https://doi.org/10.1007/s10719-013-9473-y (2013).
12. Blanco, R. *et al.* Immunodetection of N-glycolyl GM3 ganglioside in lung carcinoma by immunohistochemistry: a technical study using frozen and formalin-fixed and paraffing-embedded tissues. *Acta Microscopica* **23**, 199-213 (2014).
13. Rojas, G., Pupo, A., Gomez, S., Krengel, U. & Moreno, E. Engineering the binding site of an antibody against N-glycolyl GM3: from functional mapping to novel anti-ganglioside specificities. *ACS Chem Biol* **8**, 376-386, https://doi.org/10.1021/cb3003754 (2013).
